# Visual height intolerance and acrophobia: distressing partners for life

**DOI:** 10.1007/s00415-016-8218-9

**Published:** 2016-07-06

**Authors:** Hans-Peter Kapfhammer, Werner Fitz, Doreen Huppert, Eva Grill, Thomas Brandt

**Affiliations:** 1 Department of Psychiatry and Psychotherapeutic Medicine, Medical University of Graz, Auenbruggerplatz 31, 8036 Graz, Austria; 2 Institute for Clinical Neurosciences and German Dizziness Center, Ludwig-Maximilians University, Munich, Germany; 3 Institute for Medical Information Processing, Biometrics and Epidemiology (IBE), German Dizziness Center, Ludwig-Maximilians University, Munich, Germany

**Keywords:** Visual height intolerance, Acrophobia, Course, Social impairment, Help-seeking behavior

## Abstract

The course of illness, the degree of social impairment, and the rate of help-seeking behavior was evaluated in a sample of individuals with visual height intolerance (vHI) and acrophobia. On the basis of a previously described epidemiological sample representative of the German general population, 574 individuals with vHI were identified, 128 fulfilled the DSM-5 diagnostic criteria of acrophobia. The illness of the majority of all susceptible individuals with vHI ran a year-long chronic course. Two thirds were in the category “persistent/worse”, whereas only one third was in the category “improved/remitted”. Subjects with acrophobia showed significantly more traumatic triggers of onset, more signs of generalization to other height stimuli, higher rates of increasing intensity of symptom load, higher grades of social impairment, and greater overall negative impact on the quality of life than those with pure vHI. An unfavorable course of illness in pure vHI was predicted by major depression, agoraphobia, social phobia, posttraumatic stress, initial traumatic trigger, and female sex; an unfavorable course in acrophobia was predicted by major depression, chronic fatigue, panic attacks, initial traumatic trigger, social phobia, other specific phobic fears, and female sex. Help-seeking behavior was astonishingly low in the overall sample of individuals with vHI. The consequences of therapeutic interventions if complied with at all were quite modest. In adults pure vHI and even more so acrophobia are by no means only transitionally distressing states. In contrast to their occurrence in children they are more often persisting and disabling conditions. Both the utilization of and adequacy of treatment of these illnesses pose major challenges within primary and secondary neurological and psychiatric medical care.

## Introduction

Visual height stimuli that involve a critically large distance between the nearest visible stationary contrasts within the field of vision and the observer’s eyes may cause impaired visual control of postural balance. Three different conditions may be distinguished in this specific eliciting situation: a physiological visual height imbalance that concerns everybody, a visual height intolerance (vHI) that comprises symptoms of vertigo of varying distressing intensity, and acrophobia (height phobia) defined as a specific phobia with symptoms of a panic attack leading to avoidance behavior and psychological and/or psychosocial impairment [1]. A first population-based cross-sectional epidemiological study showed that the life-time prevalence of vHI was 28 % in the general adult population (women 32 %; men 25 %) [2]. In a study on children 8–10-year-old, vHI was also reported by more than one third of the prepubertal boys and girls without any gender preponderance. In contrast to the adult-onset type of the condition vHI in children appeared to take a benign spontaneous course [3]. A subsequent study on a large sample of adults representative of the German population almost exactly replicated the life-time prevalence of vHI (28.5 %; women 32.4 %, men 24.5 %). Fearfulness was the most prominent symptom of vHI; it worsened to panic attacks in some 22 %. According to DSM-5, the life-time prevalence of acrophobia was 6.4 % (women 8.6 %, men 4.1 %), and the point prevalence was 2.0 % (women 2.8 %; men 1.1 %). VHI and even more so acrophobia were associated with high rates of comorbid anxious and depressive conditions [4]. The life-time prevalence of acrophobia in this study was somewhat higher than in other studies, in which values of 1.9 [5], 3.1 [6], 4.9 [7], to 5.3 % were reported [8].

In general, there are several oddities about specific phobias. They are highly frequent in a community [9], often show a low rate of spontaneous symptomatic remission, and are associated with substantial impairment and other comorbid conditions [10–12]. Despite these negative aspects, only a minority of those affected actually seek professional help [13].

Based on the data set of our previous study [4], the objective of this investigation was twofold: (1) to describe the course of illness, grade of impairment, and help-seeking behavior of subjects with pure vHI and of those with acrophobia, and (2) to determine the prognostic variables of each illness.

## Methods

### Sample

A case–control study was nested within a population-based cross-sectional telephone survey (for more details on methods: [4]). A sample of 2012 individuals (men 904, women 1108) aged 14 and above, and representative of the German population was selected.

A case was defined as any participant in the survey who reported having had life-time visual height intolerance (answering yes to the question “Have you ever experienced visual height intolerance, an unpleasant feeling caused by visual exposure to heights?”). This question was further elaborated by stating that some people when on mountains, bridges, towers or similar places experience the exposure to heights as distressing and may react with unpleasant bodily or anxious feelings. They were asked whether they remembered any such event ever occurring previously in their life. If they answered “yes”, they were asked to describe this event in their own words. As a rule, no one had any difficulty understanding what the screening question was referring to. A total of 574 cases out of 2, 012 participants were identified. Controls were selected randomly from the group of participants who did not report having ever had any visual height intolerance. This approach was chosen to minimize the selection bias of the controls. The 702 controls were determined by randomization. The study interview was performed by well-trained interviewers.

### Measures

The survey questionnaire asked about sociodemographic variables (i.e., age, gender, household size, income, occupation, education). Age was stratified into seven categories: 14–20, 21–30, 31–40, 41–50, 51–60, and >60. The survey further identified prevalent symptoms of vHI, typical triggering stimuli, the course of symptoms, incidences of generalization to other eliciting visual height stimuli, psychosocial impairment and help-seeking behavior. The vHI symptom-related negative impact on life quality was measured on a visual analog scale ranging from 0: no negative impact to 100: maximal negative impact.

Acrophobia was diagnosed in individuals with vHI according to the DSM-IV-TR criteria of a specific phobia that is essentially identical to those of DSM 5, including height-related panic symptoms, persisting avoidance behavior and psychological and/or psychosocial impairment. The life-time prevalence of any anxiety condition was assessed on the level of syndromes outlined in SCID according to DSM-IV-TR (i.e., criteria of time and negative interference with daily activities or social performance were not assessed separately). In particular, any other specific phobic fears, panic attacks, agoraphobia, social phobia, general anxiety, posttraumatic stress, obsessive–compulsive or hypochondriac symptoms were ascertained. Chronic fatigue was also determined but only on the level of a syndrome agreeing with a prototypical description, i.e., without the detailed additional criteria established in the medical subspecialties for the level of disorder. Finally, a major depressive disorder was assessed according to the diagnostic criteria of DSM-IV-TR.

### Statistical analysis

Means were used to determine continuous variables and percentages for categorical variables. Explorative *t* tests and Pearson’s Chi-square tests were calculated to compare individuals with pure vHI (i.e., without symptoms qualifying for acrophobia; *n* = 446) vs individuals with acrophobia (*n* = 128). Multiple regression analyses were applied to determine any prognostic variables as regards chronicity and intensity of the symptoms in the course of illness. The following variables were included: migraine, Meniere’s disease, motion sickness susceptibility, other vertiginous diseases, major depression, chronic fatigue, alcohol consumption, panic attacks, agoraphobia, social phobia, generalized anxiety, other specific phobic fears, obsessive–compulsive symptoms, hypochondriac symptoms, posttraumatic stress symptoms (not related to height), female sex, and traumatic trigger of onset of vHI or acrophobia. For logistic regressions, IBM SPSS Statistics version 20 (IBM Corporation) was used. Statistical significance was set at the conventional two-tailed 5 % level.

## Results

### Description of clinical characteristics of the total sample with visual height intolerance

Our previous study reported the following findings [4]. The overall life-time prevalence of vHI in the total sample was 28.5 %. It was higher in women than in men (32.4 vs 24. %; *χ*^2^ = 15.4; *df* = 1; *p* < 0.001). The point prevalence of vHI was 6.4 % (women 7.6 % vs men 5.2 %; *χ*^2^ = 4.5; *df* = 1; *p* < 0.001). Individuals with vHI reported that initial height-related symptoms had occurred most often (36 %) in the second decade (*χ*^2^ = 97.3042; *df* = 6; *p* < 0.001). VHI was experienced with the intensity of panic attacks by 22.5 % (women 26.7 %, men 16.6 %; *χ*^2^ = 8.23; *df* = 1; *p* = 0.004). The overall life-time prevalence of acrophobia was calculated to be 6.4 %. It was statistically and significantly lower in men (4.1 %) than in women (8.6 %; *χ*^2^ = 17.67; *df* = 1; *p* < 0.001). The point prevalence of acrophobia was 2.0 % overall, 1.1 % in men, and 2.8 % in women (*χ*^2^ = 7.5; *df* = 1; *p* = 0.006) at the time of the interview. Table 1 presents a summary survey of the comorbidities established for the subgroups of individuals without vHI, with pure vHI, and with acrophobia. Comparisons were calculated for individuals with vHI vs those without vHI, and for individuals with pure vHI vs those with acrophobia. The latter comparison served as the basis for further analysis of the course of illness, social impairment, and help-seeking behavior. In general, the pronounced amount of comorbid affective and anxious conditions, both in individuals with vHI and even more so in those with acrophobia, had to be stressed.

**Table 1:** Survey on the comorbidities of individuals with no visual height intolerance, pure visual height intolerance, and acrophobia

	Non-vHI (*n* = 702)	Pure vHI (*n* = 445)	Acrophobia (*n* = 129)	Sum	Pure vHI + acrophobia vs non-vHI			Pure vHI vs acrophobia		
					Chi-square	*df*	*p*	Chi-square	*df*	*p*
Any anxiety condition	178	166	64	408	31.431	1	**<0.001**	6.310	1	**0.012**
Major depression	109	135	48	292	47.862	1	**<0.001**	2.221	1	0.136
Chronic fatigue	69	47	19	135	.839	1	0.360	1.765	1	0.184
Panic attacks	65	75	47	187	35.749	1	**<0.001**	22.005	1	**<0.001**
Agoraphobia	14	15	12	41	7.473	1	**0.006**	7.591	1	**0.006**
Social phobia	27	35	15	77	13.217	1	**<0.001**	1.639	1	0.200
Generalized anxiety	42	46	18	106	11.110	1	**0.001**	1.185	1	0.276
Specific phobic fears	32	49	25	106	27.917	1	**<0.001**	5.943	1	0.015
Obsession/compulsion	12	24	11	47	17.173	1	**<0.001**	1.598	1	0.206
Hypochondria	7	18	4	29	11.423	1	**0.001**	0.272	1	0.602
Posttraumatic stress	32	42	22	96	19.777	1	**<0.001**	5.546	1	0.019
Migraine	180	109	56	345	1.366	1	0.242	17.215	1	**<0.001**
Motion sickness	165	122	35	322	2.364	1	0.124	0.008	1	0.927
Menière’s disease	8	9	5	22	3.059	1	0.080	1.403	1	0.236
Other vertiginous diseases	73	28	11	112	5.325	1	**0.021**	0.818	1	0.366
Traumatic trigger of vHI		49	26	75				7.615	1	**0.006**

Bold represents that statistical significance set at the conventional two-tailed 5 % level

### Course of illness in individuals with pure visual height intolerance vs those with acrophobia

Climbing a tower was the first and most common precipitating stimulus in the overall sample of vHI, followed by hiking, climbing a ladder, walking over a bridge, working on the roof of a building, or sitting in a Ferris wheel [4]. There was no significant difference between the subgroups of pure vHI vs acrophobia as regards this initial triggering situation. Thirteen percent of individuals reported that their first occurrence of vHI had been elicited in a situation that could be considered traumatic, e.g., being rescued from a mountain in bad weather, nearly falling from the top of a roof, climbing a high tree and suddenly falling down to the ground [4]. Traumatic triggers were significantly more frequent in the subgroup of acrophobia vs pure vHI (*χ*^2^ = 7.615; *p* = 0.006). Subsequently, nearly half (47 %) of the total sample with vHI noticed that other visual height stimuli elicited typical symptoms of vHI as well. As a rule, the majority of affected individuals responded by avoiding typical triggering height stimuli. However, at least some 36 % succeeded in inventing various strategies of self-confrontation that finally contributed to improving or even overcoming vHI. The generalization tendency was significantly more pronounced in the subgroup of acrophobia vs pure vHI (*χ*^2^ = 12,971; *p* < 0.001).

**Fig. 1:**
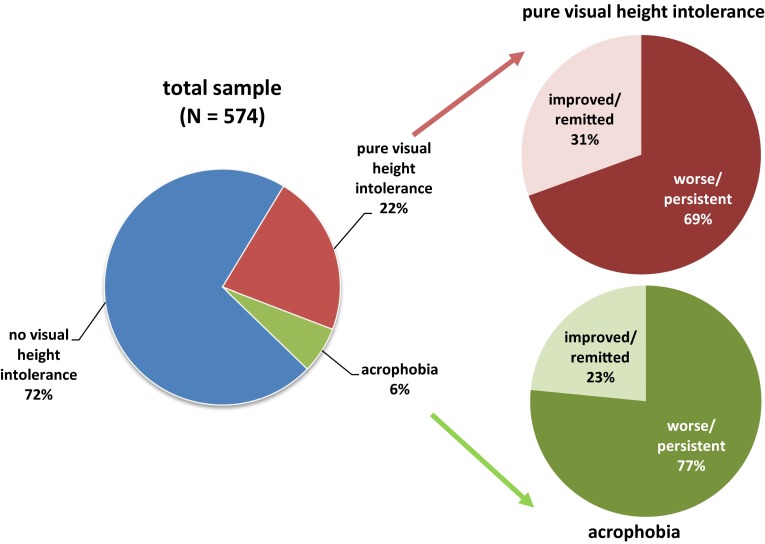
Course of illness in individuals with pure visual height intolerance (*n* = 446) vs. acrophobia (*n* = 128)

As regards the course of illness in general, after the first occurrence of vHI symptoms the illness spontaneously improved in 31 % of individuals, persisted on a constant level of intensity in 52 %, and worsened in 13 % in the further course (4 % could not report on the course of vHI symptoms [4]. Figure 1 gives an overview comparing the general course of illness in the subgroups of pure vHI vs acrophobia. Although individuals with pure vHI seemed to have a more favorable course overall (improved/remitted: 31 %; worse/persistent: 69 %) than individuals with acrophobia (improved/remitted: 23 %; worse/persistent: 77 %), this tendency did not reach the level of statistical significance set in advance (*χ*^2^ = 2452; *p* = 0.071). If the rates of increasing intensity of symptom load during the course were measured, a significantly more unfavorable development was observed in individuals with acrophobia vs pure vHI (*χ*^2^ = 4.659; *p* = 0.023).

In general, about 22 % of individuals with vHI reported that their daily activities and psychosocial role performance were still or had been seriously impaired by these symptoms [4]. Subjects with acrophobia indicated a higher grade of social impairment than those with pure vHI (*χ*^2^ = 8.71; *p* = 0.004). The overall negative impact on the quality of life in the entire sample of individuals with vHI was assessed at 42.5 on the visual analog scale [4]. Again, individuals with acrophobia showed significantly higher mean scores than those with pure vHI (mean 53 vs 34; *F* = 5.743; *p* = 0.027).

### Prognostic variables for the course of illness in individuals with pure visual height intolerance vs those with acrophobia

A predominantly unfavorable course of illness was considered over the entire sample of subjects with vHI from two perspectives. First, the general course was differentiated into the categories “improved/remitted” vs “worse/persistent”. Then the intensity of symptom load during the evolving course was measured by the rate of gradual development of “increased symptom load”. As the course of illness of subjects with pure vHI and of those with acrophobia might have run along pathways characterized by different prognostic variables, multiple regression analyses were calculated for the subgroups separately for both “chronicity” and “intensity” (Table 2). A statistically significant but more unfavorable general course was predicted for the subgroup of pure vHI on the basis of the following variables: major depression, agoraphobia, social phobia, posttraumatic stress, traumatic trigger of onset of vHI, and female sex. The prognostic variables for a higher symptom load in this subgroup included major depression, chronic fatigue, agoraphobia, posttraumatic stress, and traumatic trigger. In the subgroup of acrophobia, the corresponding prognostic variables for “chronicity” were major depression, panic attacks, traumatic trigger of onset of acrophobia, and for “intensity”, social phobia, other specific phobic fears, and female sex.

**Table 2:** Prognostic variables regarding unfavorable course of illness in individuals with pure visual height intolerance vs with acrophobia

Unfavorable course (worse/persistent)			Higher intensity of symptom load		
Pure visual height intolerance					
Prognostic variables					
Major depression	*p* = 0.008	OR 2.39	Major depression	*p* = 0.047	OR 2.02
Agoraphobia	*p* = 0.017	OR 5.46	Chronic fatigue	*p* = 0.045	OR 5.90
Social phobia	*p* < 0.001	OR 15.99	Agoraphobia	*p* = 0.006	OR 7.38
Posttraumatic stress	*p* = 0.046	OR 2.68	Posttraumatic stress	*p* = 0.036	OR 3.03
Traumatic trigger	*p* = 0.002	OR 3.53	Traumatic trigger	*p* = 0.002	OR 3.40
Female sex	*p* = 0.001	OR 2.91			
Acrophobia					
Prognostic variables					
Major depression	*p* = 0.020	OR 3.14	Social phobia	*p* = 0.022	OR 12.02
Panic attacks	*p* = 0.028	OR 9.19	Specific phobic fears	*p* = 0.003	OR 25.17
Traumatic trigger	*p* = 0.009	OR 4.75	Female sex	*p* = 0.040	OR 8.30

### Help-seeking behavior in individuals with pure visual height intolerance vs those with acrophobia

In the entire sample of individuals with vHI only 14.3 % (men 9.5 %; women 17.7 %; *χ*^2^ = 7.63; *p* = 0.006) sought some kind of help for their symptoms of vHI. Only 9 % (men 12.2 %; women 6.9 %; *χ*^2^ = 4.53; *p* = 0.033) contacted a medical doctor. The rate of medical consultations for vHI was highest (19 %) for the age group over 60 years, and most often general practitioners were contacted (78 %) followed by neurologists (40 %), ENT specialists (38 %), and psychiatrists (33 %). The usual treatment consisted of either some kind of medication (55 %) or unspecific behavioral advice, e.g., how to confront the problem (46 %). The overall consequence of these medical interventions was rather modest. About two thirds of those seeking help experienced no change in vHI symptoms, although women seemed to benefit better from their therapeutic contacts (45.5 %) than men (14.8 %; *χ*^2^ = 5.58; *p* = 0.018). There seemed to be a somewhat higher chance of gaining some symptomatic improvement if medication was combined with some psychological intervention as opposed to only medication, but this trend did not reach the level of statistical significance (*χ*^2^ = 1827; *p* = 0.157).

Overall, subjects with acrophobia sought help significantly more frequently than those with pure vHI (*χ*^2^ = 12,984; *p* < 0.001). Acrophobic individuals were treated more often with medication and psychological intervention than with medication alone, whereas individuals with pure vHI predominantly received only medication (*χ*^2^ = 16,095; *p* < 0.001). However, the benefit from the kind of therapy received was not significantly different between the two subgroups (*χ*^2^ = 0.148; *p* = 0.487).

## Discussion

### Predominantly chronic course of illness

The focus of the investigation was on a more detailed description of the course of illness in subjects who reported having experienced a distressing event of visual height intolerance.

The majority of individuals with vHI had a year-long chronic course of illness; roughly two thirds had a “persistent/worse” course and only one third an “improved/remitted” course. The overall tendency for symptoms to persist was high, since remission, if it finally occurred, took many months or years even. The basic characteristics of the course of illness of subjects with acrophobia compared to those with pure vHI showed statistically and significantly more traumatic triggers of onset, more signs of generalization to other height stimuli, higher rates of increasing intensity of symptom load, higher grades of social impairment, and more overall negative impact on the quality of life. This finding agrees well with other empirical observations made on specific phobias in general, and acrophobia in particular [9, 10, 14]. It however differs from an earlier study on primary school children aged 8–10 years which revealed a slightly higher susceptibility rate to vHI (34 %) compared to the prevalence rate of 28 % in adults [3]. This seemingly contradicts the data of our earlier study in adults, in which only 4.5 % of susceptible individuals indicated that their first manifestation of vHI occurred in the first decade [4]. The most probable explanation for this is that intolerance to heights in children takes a benign spontaneous course with remission within a few years. This view is supported by the interviews in which nearly half of the children reported a spontaneous improvement or remission already at an age of 8–10 years [3].

### Differential variables predicting chronicity and intensity of the course of illness

In our previous study, we discussed the intricate interrelationships of vHI in general and of pure vHI and acrophobia in particular with comorbid neurological, anxiety, and depressive conditions. These interrelationships were further analyzed by their primary and secondary manifestations, i.e., what predicted what in the time course [4]. Tab. 1 of this study underlined once more that differential comorbid conditions were found in the overall comparison of individuals without vHI vs with vHI as well as in the special comparison of individuals with pure vHI vs acrophobia. The latter comparison revealed that subjects with acrophobia showed more pronounced comorbid agoraphobia, panic attacks (other than those elicited by height stimuli), more anxiety, and more migraine compared to subjects with pure vHI. We generally assumed that comorbidity quite likely would have a major negative impact on the course of illness. However, we had to consider the possibility that variables making up comorbid conditions as such in both pure vHI and acrophobia might be different from those variables determining a more unfavorable course for both pure vHI and acrophobia. Therefore, for all variables listed in tab. 1, we calculated multiple regression analyses to define those variables that significantly predicted a more unfavorable course of illness as “persistent/worse” and also determined the rate of a developing “increased intensity of symptom load”. We performed multiple regression analyses to assess “chronicity” and “intensity” for the subgroups of pure vHI and acrophobia separately.

Both a chronic general course and a higher intensity of symptom load were quite consistently predicted by the variables major depression, chronic fatigue, agoraphobia, social phobia, posttraumatic stress, and initial traumatic trigger in individuals with pure vHI. Female sex was prognostic for only chronicity. The pattern of prognostic variables for individuals with acrophobia differed: major depression, panic attacks, and initial traumatic trigger predicted chronicity, whereas social phobia, other specific phobic fears, and female sex predicted intensity.

Secondary depression may be considered a general feature, even though an outstanding indicator of chronicity in the course of any primary mental and physical disorder, above all in anxiety and stress-related disorders [15, 16]. From a clinical point of view “physiological height imbalance”, “pure visual height intolerance” and “acrophobia” may be considered distinct variants of psychophysiological reactivity to height stimuli. They seem to lie on a continuum ranging from normal physiological to ever more distressing reactions, and finally evolve into a specific phobia with panic attacks in circumscribed height situations [1, 17]. This assumed continuum seems to reasonably explain the variants of pure vHI and acrophobia which differ only by varying rates of panic attacks. Actually, however, it was not only a matter of increasing symptom severity in both conditions. The multiple regression analyses showed that in acrophobia the very fact of a specific phobic fear might have contributed a unique qualitative psychopathological and psychobiological dimension which mediated differential variables predicting an unfavorable course of illness compared to those prognostic variables calculated for pure vHI. This is in line with the empirical fact that specific phobias, in general, probably occupy a discrete nosological position within the group of anxiety disorders [18, 19]. In terms of etiology, the acquisition of height-related symptoms in both vHI and acrophobia may best be explained to be a consequence of non-associative learning, i.e., a mode of evolution-primed response rather than traumatically induced classic conditioning [20–22]. In terms of the course of illness, however, initial traumatic triggers made an independent contribution to a more chronic and more intensive course in both pure vHI and acrophobia.

### Extremely low help-seeking behavior and even more modest overall treatment results

One of the most bewildering findings of our study was that despite an overwhelmingly chronic course of illness, major psychiatric comorbidity and remarkable social impairment in both pure vHI and even more so in acrophobia, the rate of seeking medical help was quite low: only 14 % of the entire sample of vHI, somewhat more in women than in men. This observation definitely agrees with findings in the literature on general population-based samples of subjects with phobic fears in general and with height fears in particular [13, 14, 23]. Nevertheless, further empirical exploration is needed, since unfortunately the data set collected in this study could not provide more specific details. Even in the small minority of subjects who actually sought professional help during their often year-long course of height-related complaints, only the variables acrophobia vs pure vHI, i.e., higher chronicity and intensity of symptom load, and female sex correlated positively with help-seeking behavior. It was no surprise that general practitioners had been contacted most often, distinctly less often neurologists, ENT specialists, and least of all psychiatrists. The kind of treatment these individuals received included some medication, best when combined with some unspecific behavioral advice or confrontational technique. The consequences were that two thirds of help-seeking subjects did not receive any symptomatic benefit from the therapeutic interventions. The overall sobering observation is that help-seeking behavior of these patients is generally low and the treatment inadequate under usual conditions in primary and secondary medical care. Our finding, however, agrees with corresponding data on treatment realities for anxiety disorders in general [24].

### Prospect

Several major demands must now be made for better treatment of individuals with vHI and acrophobia. Both illnesses are affected by height-related troubles, and therefore, general practitioners, neurologists, ENT specialists, and psychiatrists should be made more aware that vHI and acrophobia are not only highly prevalent in the general population, but are also associated with significantly increased risks of comorbid anxiety and depressive disorders. VHI is by no means only transitionally distressing; it is more often a persisting and disabling condition that requires adequate treatment, at least when it first manifests in adulthood. This is especially true for acrophobia [2, 4]. Proper psychoeducation should elucidate the various neurophysiological characteristics of individuals with vHI and acrophobia, for example, the recently reported typical restrictions of visual exploration and imbalance exhibited during stance and locomotion when such individuals are exposed to heights [25, 26]. These findings could also be quite beneficially integrated into a therapeutic approach based on behavioral techniques applied during real confrontations or virtual reality exposure [1]. This conceptually promising approach, however, still requires validation by randomized controlled trials. In general, two cross-sectional epidemiological studies, one with 3517 subjects and one with 2012 individuals showed that only 11 %, respectively, 14 % of those susceptible to vHI had consulted a doctor, most often a general practitioner, followed by neurologists, otolaryngologists, and psychiatrists. The diagnosis of fear of heights or vHI had initially been medically established in only less than 25 % [2, 17]. We therefore conclude that these medical conditions should be added to the diagnostic spectrum of neurologists and psychiatrists. Unfortunately, they have hitherto been largely neglected in the training curricula of both specializations as well as in the relevant textbooks.
